# Bioinformatics analysis reveals novel tumor antigens and immune subtypes of skin cutaneous melanoma contributing to mRNA vaccine development

**DOI:** 10.3389/fimmu.2025.1520505

**Published:** 2025-02-24

**Authors:** Ronghua Yang, Jia He, Deni Kang, Yao Chen, Jie Huang, Jiehua Li, Xinyi Wang, Sitong Zhou

**Affiliations:** ^1^ Department of Burn and Plastic Surgery, Guangzhou First People’s Hospital, Guangzhou Medical University, Guangzhou, Guangdong, China; ^2^ Department of Burn Surgery, The First People’s Hospital of Foshan, Foshan, Guangdong, China; ^3^ Department of Dermatology, The First People’s Hospital of Foshan, Foshan, Guangdong, China

**Keywords:** bioinformatics analysis, skin cutaneous melanoma, mRNA vaccine, tumor antigens, immune subtypes, immune landscape

## Abstract

**Introduction:**

Skin cutaneous melanoma (SKCM) is a common malignant skin cancer with high mortality and recurrence rates. Although the mRNA vaccine is a promising strategy for cancer treatment, its application against SKCM remains confusing. In this study, we employed computational bioinformatics analysis to explore SKCM-associated antigens for an mRNA vaccine and suitable populations for vaccination.

**Methods:**

Gene expression and clinical data were retrieved from GEO and TCGA. The differential expression levels and prognostic index of selected antigens were computed via GEPIA2,while genetic alterations were analyzed using cBioPortal. TIMER was utilized to assess the correlation between antigen-presenting cell infiltration and antigen. Consensus clustering identified immune subtypes, and immune characteristics were evaluated across subtypes. Weighted gene co-expression network analysis was performed to identify modules of immune-related genes.

**Results:**

We discovered five tumor antigens (P2RY6, PLA2G2D, RBM47, SEL1L3, and SPIB) that are significantly increased and mutated, which correlate with the survival of patients and the presence of immune cells that present these antigens. Our analysis revealed two distinct immune subtypes among the SKCM samples. Immune subtype 1 was associated with poorer clinical outcomes and exhibited low levels of immune activity, characterized by fewer mutations and lower immune cell infiltration. In contrast, immune subtype 2 showed higher immune activity and better patient outcomes. Subsequently, the immune landscape of SKCM exhibited immune heterogeneity among patients, and a key gene module that is enriched in immune-related pathways was identified.

**Conclusions:**

Our findings suggest that the identified tumor antigens could serve as valuable targets for developing mRNA vaccines against SKCM, particularly for patients in immune subtype 1. This research provides valuable insights into personalized immunotherapy approaches for this challenging cancer and highlights the advantages of bioinformatics in identifying immune targets and optimizing treatment approaches.

## Introduction

Skin cutaneous melanoma (SKCM) is the most lethal and aggressive form of cutaneous malignancy, with a rapidly increasing incidence rate worldwide (1). It accounts for more than 80% of skin cancer deaths, and the primary environmental risk factor for its development is ultraviolet (UV) (2). Currently, the availability of standard treatment options for SKCM includes surgical resection, chemotherapy, and radiation therapy. The 5-year survival for localized SKCM is 99%, but it decreased to 20% when the cancer cells have metastasized. Recently, treatment options for patients with metastatic SKCM have significantly improved by application with immune checkpoint blockade and targeted therapies (BRAF and MEK kinase inhibitors) (3). Despite emerging new diagnosis and treatments, the clinical outcome of SKCM patients is still poor, with severe side effects and a high risk of drug resistance (4, 5). Thus, new and precise therapeutic methods are needed to improve the prognosis of SKCM patients.

In recent years, immunotherapies for SKCM, including Cytotoxic T Lymphocyte Antigen-4 (CTLA-4) blockade, Programmed death-1 (PD-1) blockade, talimogene laherparepvec (T-VEC), cytokines, vaccines, adoptive T cell transfer, and other strategies, have increased rapidly (6). SKCM is an ideal immunotherapeutic candidate because of its high level of lymphocytic infiltration in both primary lesions and metastatic sites (7). In addition, patients with metastatic SKCM have large heterogeneous tumor microenvironments, resulting in their heterogeneities of response to immunotherapy (8). Hence, SKCM is sensitive to immune modulation.

Among tumor immunotherapies, tumor vaccines are full of potential and attractive. Therapeutic tumor vaccines are designed to stimulate the immune system against specific tumor antigens to control tumor progression, efficiently eliminate cancer cells, develop adequate antitumor immunological memory, and prevent non-specific or adverse reactions (9). According to the antigen form, tumor vaccines are classified into peptide, tumor cell, dendritic cell, DNA, and RNA type (10). With significant technological development, mRNA vaccines represent a promising alternative to conventional vaccine approaches due to their high efficacy, safety profile, easy manufacture, and safe administration (11). The mRNA tumor vaccines are usually prepared by the template mRNA of translated proteins. After injection into the body, mRNA tumor vaccines activate the protein synthesis system of human cells to synthesize specific antigen protein, which followed induces the immune response and targets tumor cells (12). The mRNA vaccines cause innate and adaptive immune responses to enhance the antitumor effects of B and T cells. In the immune cells, exogenous mRNA activates innate immune response through Toll-like receptors to detect pathogen-associated molecular patterns (PAMPs); In the non-immune cells, RIG-1 and MDA5 sense the exogenous mRNA and then induce an IFN I-mediated immune response. For adaptive immune response, antigen-presenting cells take up the encoded proteins of exogenous mRNA and present the antigens to CD4+ T cells, and followed promotes antitumor CD8+T cell and B cell responses (13, 14). Based on the unique characteristics exhibited by individual tumors, cancer vaccines have the capability to effectively deliver targeted therapy. In 2021, Huang et al. explored novel antigens for developing mRNA vaccines, and investigated immunotyping for identifying suitable cancer patients for vaccination (15, 16). Subsequently, researchers utilized similar methods to obtain a complex immune landscape for mRNA vaccine treatment of various cancers and define appropriate vaccination patients. Therefore, utilizing vaccination as a potential approach can address the challenges presented by tumor heterogeneity and offer personalized treatment for cancers (17).

Recently, several preclinical and clinical trials of mRNA vaccine therapeutics have been proven effective in multiple tumors, including SKCM. One mRNA vaccine based on six SKCM-specific antigens (Melan-A, MAGE-A1, MAGE-A3, Survivin, GP100, and Tyrosinase) has shown an increase of vaccine-specific T cells in phase I/II clinical trials in metastatic SKCM patients (18). Another mRNA vaccine targeting antigens (CD40L, TLR4, CD70 plus tyrosinase or MAGE-A3 or MAGE-C2 or gp100) treated recurrent SKCM patients demonstrating its favorable immune activation (19). Treated with mRNA vaccine targeting 20 tumor neoantigens, patients with resected SKCM showed the risk of recurrence/death was significantly reduced compared to pembrolizumab monotherapy (20, 21). Due to tumor heterogeneity and complex immune microenvironment, research on effective anti-SKCM mRNA vaccines and identification of suitable cancer vaccination patients remain viable.

In recent years, bioinformatics has become crucial in the identification and development of tumor vaccines. Traditional vaccine design methods are often time-consuming, costly, and have high failure rates due to the extensive research needed to identify target antigens and establish immunological correlations. In contrast, in silico approaches leverage computational tools and databases to efficiently identify promising vaccine candidates. Recent advancements in bioinformatics have facilitated the creation of vaccine candidates capable of eliciting strong immune responses against a variety of human pathogens (22). By utilizing comprehensive databases, researchers can access a wealth of experimentally validated vaccine components, which are crucial for informed vaccine design (23, 24). Moreover, high-throughput screening methods and sophisticated computational algorithms have emerged as powerful bioinformatics protocols for predicting novel vaccine candidates and identifying immune epitopes that engage immune cells effectively (25). Hence, how to utilize bioinformatics strategies to optimize vaccine development and enhance antigen selection is a promising research direction.

In this study, potential antigens associated with patient survival and infiltration of antigen-presenting cells were firstly identified for SKCM mRNA vaccines by bioinformatics analysis. SKCM patients were then classified according to immune-associated genes. The immune phenotype of each immune subtype was next determined by mutational status, expression profiles of immune checkpoints and immune cell death modulators. Following, the immune landscape of SKCM elucidated considerable heterogeneity among individual patients. Finally, WGCNA can identify key gene modules. Altogether, our findings provide a theoretical basis for mRNA vaccines development and highlights the personalized treatment strategies for SKCM.

## Methods

### Data collection and preprocessing

Data on gene expression (fragments per kilobase million, FPKM) data, genomic mutation, and the corresponding clinical features of 472 patients with SKCM were obtained from The Cancer Genome Atlas (TCGA, https://www.cancer.gov/tcga) and downloaded from UCSC Xena (https://xena.ucsc.edu/). Next, the RNA-sequencing data and clinical information of 78 SKCM samples were collected from Gene Expression Omnibus (GEO, GSE54467) database. GPL6884 probes were utilized to map the GSE54467 dataset, and gene expression was normalized by R package “limma” for further analysis.

Subsequently, a total of 2,006 immune-related genes, including immune cell-specific genes, cytokines together with their respective receptors, antigen processing and presentation-associated genes, and others were acquired from the IMMPORT database (https://immport.org). We selected immune cell death-related genes and immune checkpoint-related genes from previous publications (15, 16).

### GEPIA and survival analysis of candidate antigens

The online database Gene Expression Profiling Interactive Analysis (GEPIA2) (http://gepia2.cancer-pku.cn/), which combined the TCGA dataset and the Genotype-Tissue Expression (GTEx) database, was employed for gene expression analysis. Differentially expressed genes (DEGs) were selected by ANOVA analysis with |log2FC| >2 and q-value <0.01 and their chromosomal distribution was plotted. Based on the Kaplan-Meier method with a median cutoff, overall survival (OS) and disease-free survival (DFS) were estimated and then tested by the log-rank test. P-value < 0.05 was considered statistically significant.

### cBioPortal database analysis

The cBio cancer genomics portal (cBioPortal, http://www.cbioportal.org) database was utilized to analyze the genetic variation in TCGA-SKCM patients. Data on microsatellite instability and tumor mutation burden in SKCM were obtained. Statistical significance was determined by P-value < 0.05.

### Immune cell infiltration estimation with TIMER analysis

The relationship between potential tumor antigens and antigen-presenting cells, including B cells, macrophages, and dendritic cells, was investigated by Tumor IMmune Estimation Resource (TIMER, https://cistrome.shinyapps.io/timer/) database. Purity adjustments were performed using Spearman’s correlation analysis. P-value<0.05 was signifcant.

### Development and validation of the immune subtypes

The “ConsensusClusterPlus” R package was used to distinguish the immune subtypes according to the expression profiles of 2,006 immune-related genes. Specifically, the 1,000 bootstraps were performed with 80% patients resampling and “1-Pearson correlation” as the distance metric. a range of cluster number from 2 to 9 were tested for clustering analysis, and the optimal number was determined by evaluating the consensus matrix and the consensus cumulative distribution function (CDF). The prognostic significance of these two immune subtypes was evaluated through Kaplan-Meier survival analysis. Furthermore, we compared the distribution of tumor mutational burden (TMB), mutation counts, and microsatellite instability (MSI) between the immune subtypes. The differentially expression level of immune checkpoints and immune cell death modulators were also compared between the immune subtypes.

### Analysis of cellular and molecular characteristics in the immune subtypes

A total of 28 immune signatures representing diverse immune cell types, functions, and pathways were acquired for analysis. We employed the ssGSEA algorithm via the R package “GSVA” to comprehensively calculate the relative abundance of each immune signature within the respective SKCM samples. The ImmuneScore, StromalScore, and ESTIMATEScore were determined by the “ESTIMATE” package.

### Construction of immune landscape

According to the immune gene expression profile, the dimensionality reduction analysis using the graph learningbased method was performed by the “monocle” R package to visualize the distribution of each patient. The maximum number of components was set to 2. Then, the discriminative dimensionality reduction with trees (DDRTree) method was utilized for dimension reduction. The immune landscape was displayed with the function plot cell trajectory, and each immune cluster was plotted with a particular color. The similarity of immune status among patients was determined via the pseudo-time analysis. Moreover, we explored the correlation among 28 immune cells and individual principal components (PCA1 and PCA2) through Pearson correlation analysis, and the differences in the abundance of immune cells were calculated with the appliance of the Wilcoxon test. Survival analysis was conducted in four distinct state subtypes.

### Weighted gene co-expression network analysis

The co-expression modules of immune-related genes were obtained using the “WGCNA” R package. In order to construct a correlation adjacency matrix, the soft-thresholding power was selected according to the scale-free network topology criterion. We then recognized co-expression modules using the bottom-up algorithm and dynamic tree cut method to estimate module eigengenes (MEs) and quantify module similarity. Univariate Cox regression analysis was performed to investigate the prognostic value of different modules. Gene Ontology (GO) and Kyoto Encyclopedia of Genes and Genomes (KEGG) enrichment analysis were employed to annotate the functions of the module genes that are closely related to the immune subtypes.

### Reverse transcription quantitative real-time PCR (RT-qPCR)

The study obtained discarded normal skin tissue from two patients with skin wounds during the excision of damaged skin, along with tumor tissue from one patient diagnosed with SKCM. Total RNA was extracted from two normal skin tissues and SKCM tissue for using the Trizol reagent (Invitrogen, United States) as the manufacturer’s instructions. Reverse transcription of the obtained RNA was done using the First-Strand cDNA Synthesis Super Mix for qPCR along with a one-step gDNA remover (AmeriDx, United States). SYBR Green-based real-time qPCR was performed using the specific primer pairs for each target gene (**Supplementary Table S1**). The relative changes in gene expression were determined using the 2^-ΔΔ^ *^CT^* method.

### Statistical analysis

R software (version 4.1.1) and its corresponding R packages were performed for statistical data analysis. The Wilcox test was implemented to compare data between the two groups, while the Kruskal-Wallis test was conducted to compare three or more groups. Survival analysis performed by the Kaplan-Meier method with log-rank test and univariate Cox regression analysis. Correlations were determined by Spearman’s analysis. A two-tailed P-value < 0.05 was considered statistically significant.

## Results

### Screening potential tumor antigens of SKCM

**Figure 1:**
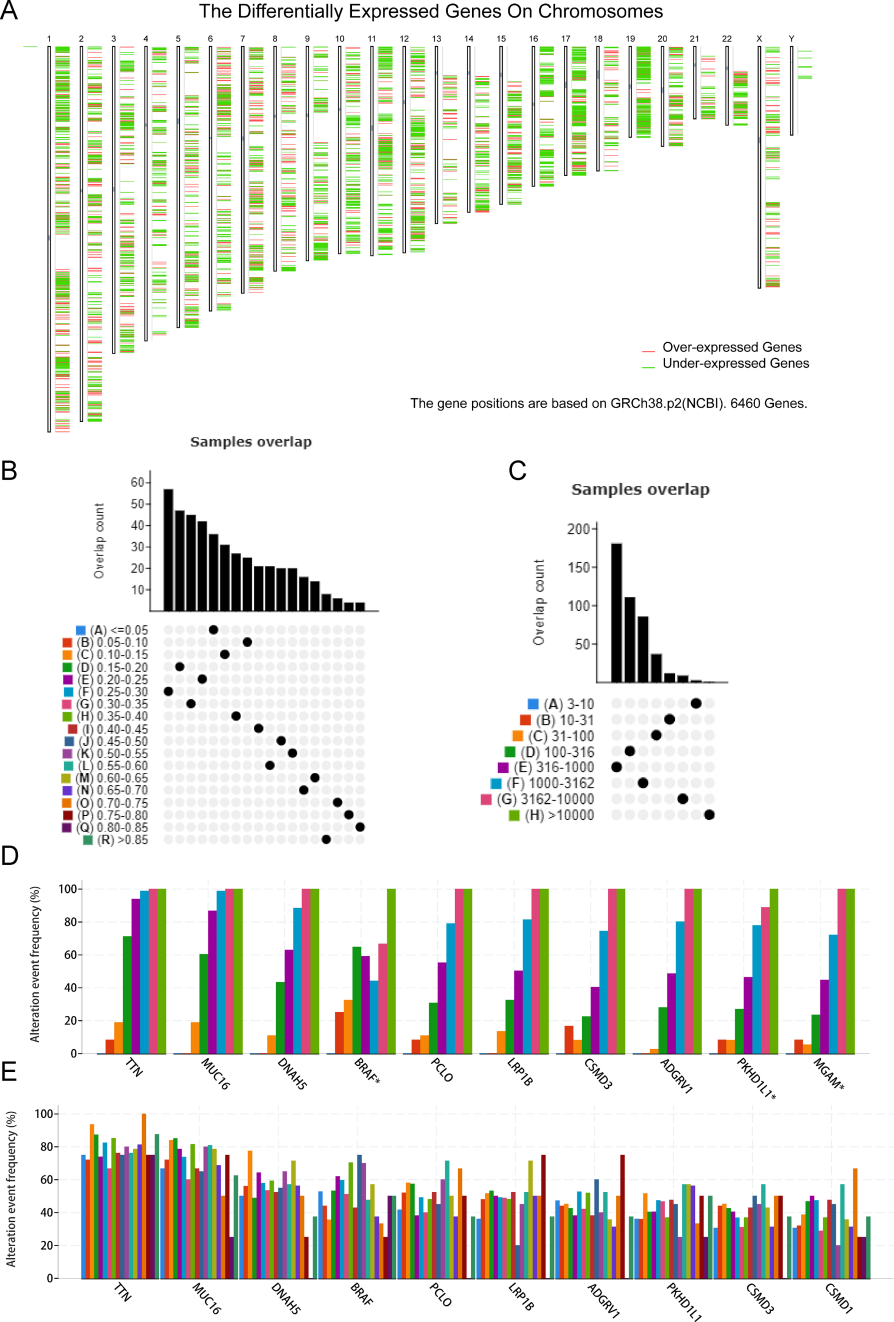
Identification of candidate neoantigens in SKCM. **(A)** Chromosomal distribution of up-and down-regulated genes in SKCM. **(B, C)** Samples overlapping in altered genome fraction **(B)** and mutation counts **(C)**. **(D, E)** Top ten genes with highest frequency in altered genome fraction **(D)** and mutation counts **(E)**.

The workflow of this research is presented in **Supplementary Figure S1**. To explore candidate tumor-specific antigens in SKCM for mRNA vaccine development, we first detected 6,460 dysregulated expressed genes. Among them, 1,109 genes potentially encoding tumor-associated antigens were unregulated (**Figure 1A**). Then, 7,364 mutated genes were selected after analyzing altered genome fraction and mutation counts in each sample (**Figures 1B, C**). Of note, genes with the highest frequency in both altered genome fraction and mutation counts were observed as TTN, MUC16, DNAH5, BRAF, PCLO, LRP1B, ADGRV1, CSMD3, PKHD1L (**Figures 1D, E**). Finally, a total of 516 genes were identified as potential target antigens through the combination analysis of the expression and mutation data of SKCM patients.

### Identification of prognosis associated SKCM tumor antigens

**Figure 2:**
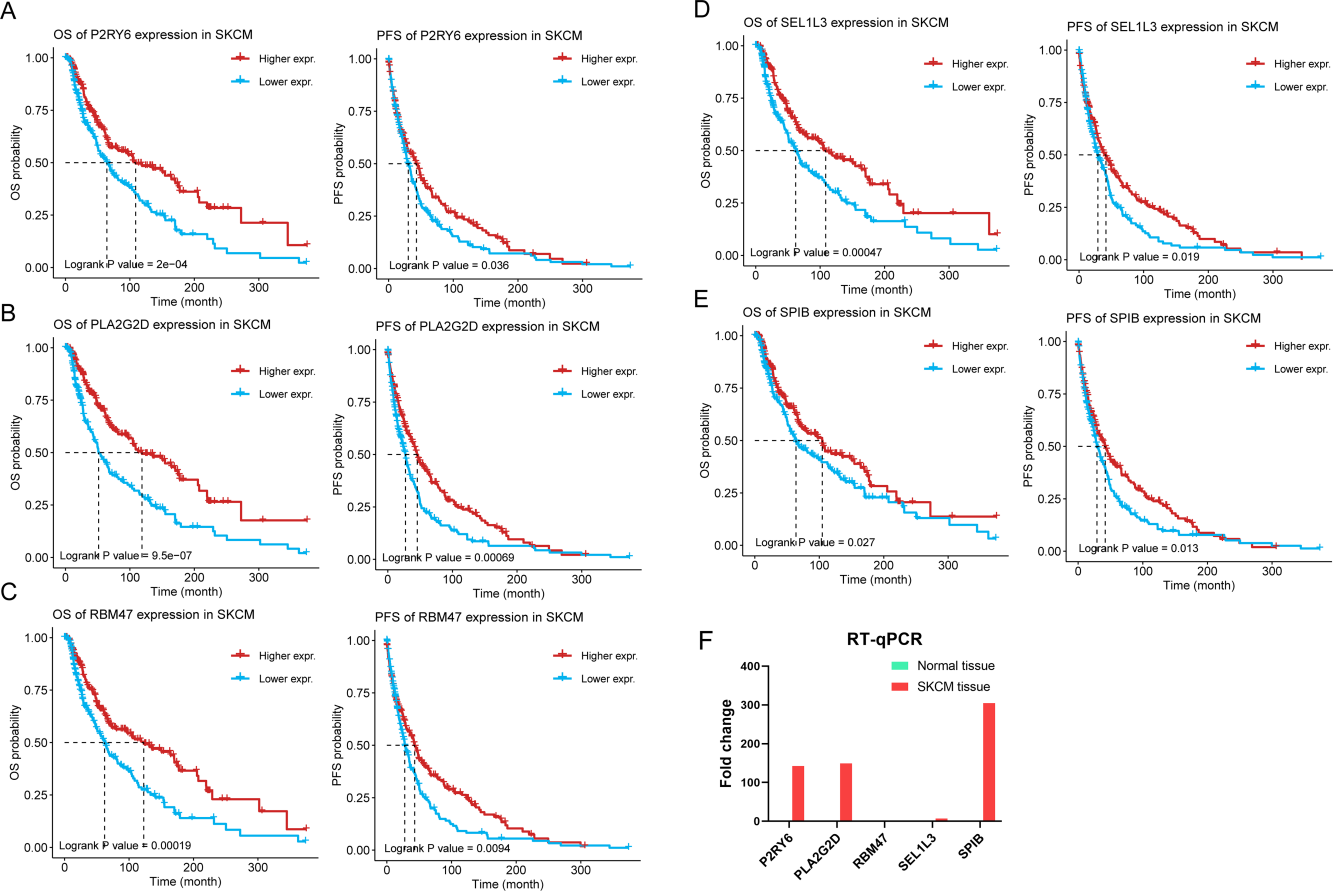
Relationships of five potential antigens with prognosis and their expression in SKCM tissue. **(A-E)** Kaplan-Meier OS and DFS curves comparing the groups with high and low expressions of P2RY6 **(A)**, PLA2G2D **(B)**, RBM47 **(C)**, SEL1L3 **(D)**, SPIB **(E)**. **(F)** RT-qPCR of SKCM and normal tissues. mRNA quantification of P2RY6, PLA2G2D, RBM47, SEL1L3, and SPIB was normalized to GAPDH.

Subsequently, the 516 candidate genes obtained above were used for survival analysis to develop prognostically relevant antigens. We discovered 109 genes closely linked to the overall survival (OS) and disease - free survival (DFS) of SKCM patients. Further analysis indicated five genes, including P2RY6, PLA2G2D, RBM47, SEL1L3, and SPIB, were positively correlated with the level of infiltration of B cells, macrophages, and dendritic cells (DCs) (**Supplementary Figure S2**). Interestingly, patients with higher expression levels of these five genes had significantly better OS and DFS than those with lower levels (**Figures 2A–E**). Hence, five potential tumorspecific antigens (P2RY6, PLA2G2D, RBM47, SEL1L3, and SPIB) were identified for developing SKCM-mRNA vaccines. Subsequently, we examined the mRNA expression of five candidate genes by analyzing normal skin tissue from the margins of skin wounds in two patients and tumor tissue from one patient diagnosed with SKCM. RT-qPCR revealed significantly elevated expression levels of P2RY6, PLA2G2D, SEL1L3, and SPIB in the SKCM tissue compared to normal tissue. These results not only validate our bioinformatics findings but also underscore the potential of these genes as biomarkers for mRNA vaccine development in SKCM (**Figure 2F**).

### Construction and validation of immune subtypes in SKCM

**Figure 3:**
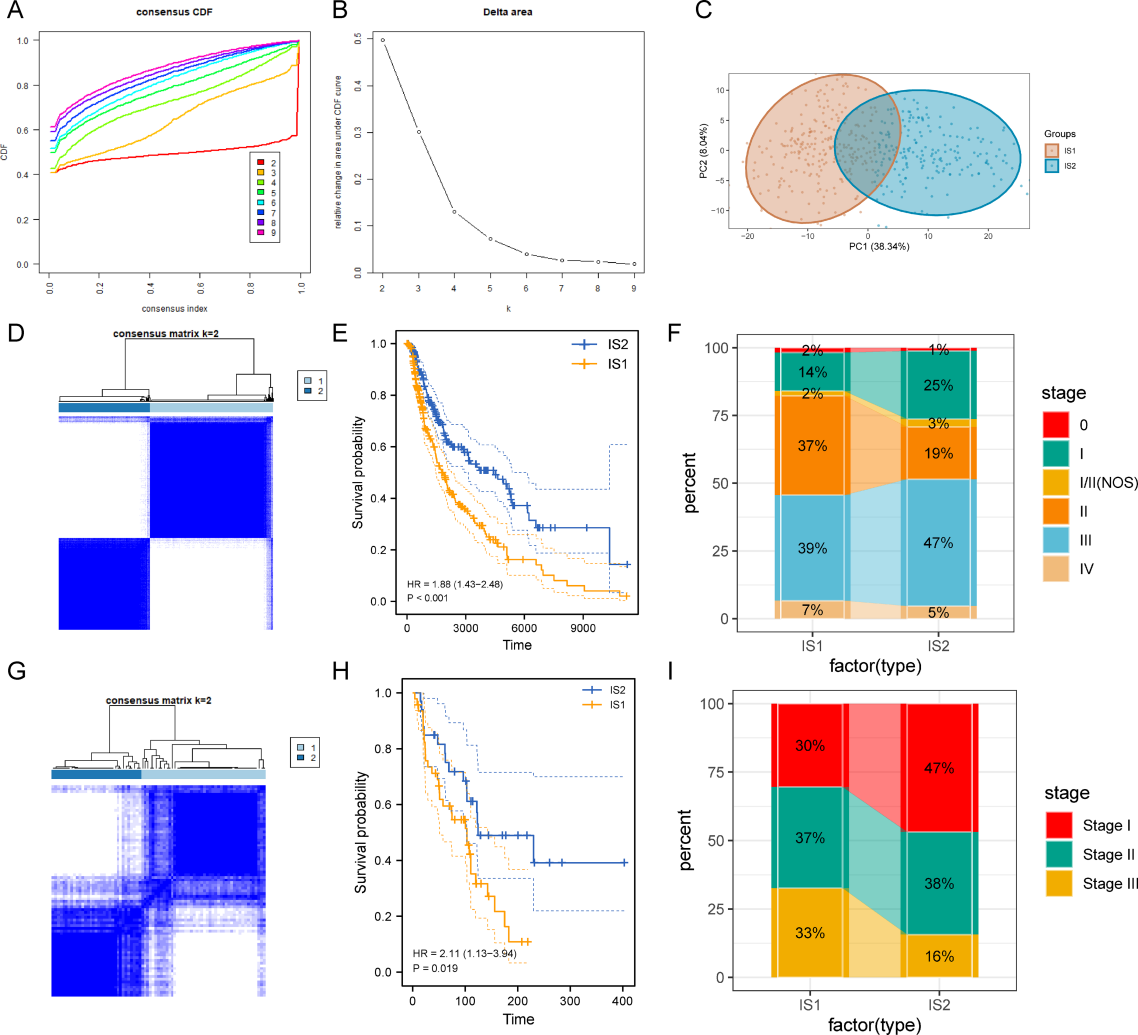
Identification and validation of immune subtypes of SKCM. **(A)** Consensus clustering CDF for k = 2 to k = 9. **(B)** delta area of immune-related genes in TCGA cohort. **(C)** Principal component analysis of the distribution of each sample in two subtypes. **(D)** Heatmap representing consensus clustering matrix of TCGA-SKCM samples for k=2. **(E)** Kaplan-Meier curves displaying OS of SKCM immune subtypes in TCGA cohort. **(F)** Different stage distribution of SKCM patients between the IS1 and IS2 immune subtypes. **(G)** Heatmap for sample clustering in the GSE54467 cohort. **(H)** Survival analysis of immune subtypes in the GSE54467 cohort. **(I)** Distribution of different stages in the two immune subtypes in the GSE54467 cohort.

Immunotyping was supported to reflect the immune status of the tumor microenvironment and thus assist in identifying suitable patients who benefit from vaccination. Therefore, consensus clustering was performed in TCGA-SKCM samples in accordance with corresponding 2,006 immune-related genes to identify different immune subtypes. The optimal cluster number was determined (k = 2) by the delta area and CFD curves; Ultimately, two immune subtypes were defined and referred as immune subtype 1 (IS1) and immune subtype 1 (IS2) (**Figures 3A, B** **).** Principal component analysis and consensus matrix revealed distinct separations between the two immune subtypes (**Figures 3C, D**). The SKCM patients in IS2 had a better survival prognosis than those in IS1 (**Figure 3E**). Further analysis of the subtype distribution of TCGA-SKCM patients with different stages indicated patients in IS1 were associated with the higher stage compared with those in IS2 (**Figure 3F**). Afterwards, the same clustering method was applied to validate in the GSE54467 cohort (**Figure 3G**). Consistent with our results obtained from the TCGA cohort, patients in IS2 had a longer survival time and a larger proportion in stage I (**Figures 3H, I**). These observations suggested that novel immune subtypes identified in this study could be used as effective prognostic biomarkers in SKCM patients.

### The mutational status in immune subtypes

Recent research demonstrated that the TMB and the frequency of somatic mutations were considered as predictors of immunotherapy response (26, 27). We calculated the TMB, mutation counts, and MSI between IS1 and IS2. Results showed that the TMB and the number of mutations in IS1 were lower than those in IS2, while there were no detectable differences in MSI (**Supplementary Figures S3A–C**). Moreover, we summarized the mutation landscape of the two subtypes, and the top 30 genes with the highest mutation frequencies were drawn in the waterfall plot (**Supplementary Figure S3D**). Together, these findings indicated that TMB and mutation counts could serve as potential indicators for mRNA vaccine treatment, and different immune subtypes exhibited specific mutational characteristics.

### Association of immune subtypes with immune modulators

Immune cell death is a type of regulated cell death that induces adaptive immunity and creates an inflamed, immunologic tumor environment (28). Immune checkpoints refer to molecules that act as gatekeepers of immune responses (29). Thus, immune checkpoints and immune cell death modulators are of remarkable importance in anticancer immunity and influence the mRNA vaccine’s efficacy. We compared the expression levels of immune checkpoints and immune cell death modulators in different immune subtypes. A total of 25 immune cell death modulators were detected in the TCGA cohort, while 21 immune cell death modulators were found in the GSE54467 cohort. The overall expression levels of these immune cell death modulators in IS2 were higher than in IS1. Notably, CXCL10, IFNAR1, FPR1, IFNAR2, and TLR4 presented the most significant differences between the immune subtypes (**Supplementary Figures S4A, B**). Furthermore, 46 and 37 immune checkpoints were detected in the TCGA and GSE54467 cohorts, respectively. The same general trends of gene expression changes of these immune checkpoints were discovered in different immune subtypes. Most selected immune checkpoints, including ADORA2A, BTLA, CD160, CD200R1, CD244, CD274 (PD-L1) and others, were highly expressed in IS2 (**Supplementary Figures S4C, D**). In conclusion, immunophenotyping reflects the expression levels of immune checkpoints and immune cell death modulators and can be taken as biomarkers for mRNA vaccines.

### Cellular and molecular characteristics of immune subtypes

**Figure 4:**
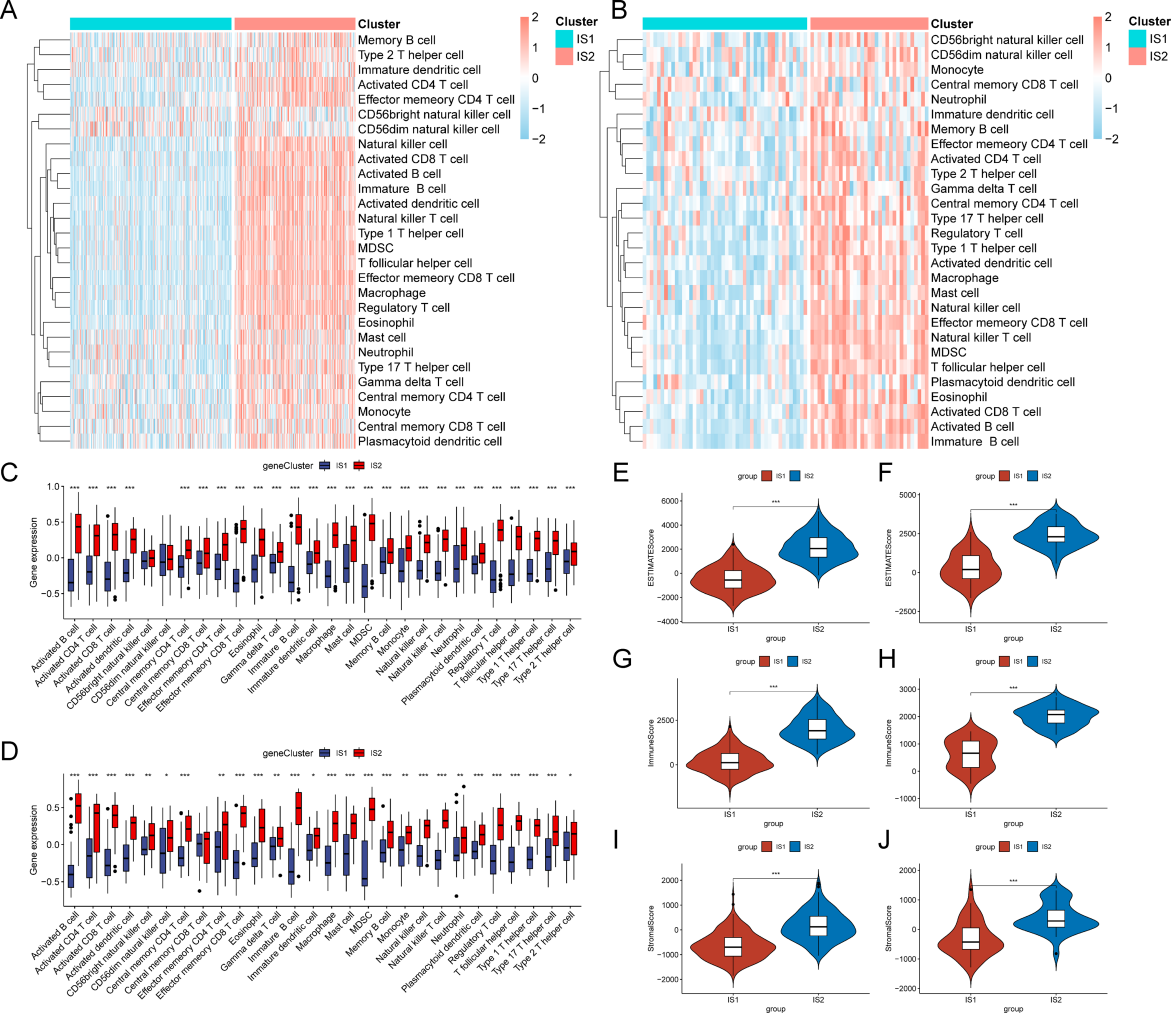
Cellular and molecular characteristics in different subtypes. **(A, B)** Heatmaps of 28 immune cell signatures between immune subtypes in the TCGA **(A)** and GSE54467 **(B)** cohorts. **(C, D)** Boxplot showing differential expression of immune cells in the TCGA **(C)** and GSE54467 **(D)** cohorts. **(E, F)** The estimate scores were assessed in the TCGA **(E)** and GSE54467 **(F)** cohorts. **(G, H)** The immune scores were evaluated by the ESTIMATE algorithm in the TCGA **(G)** and GSE54467 **(H)** cohorts. **(I, J)** Comparison of the immune scores in each immune subtype by using the ESTIMATE algorithm in the TCGA **(I)** and GSE54467 **(J)** cohorts. *p < 0.05 ,**p < 0.01 and ***p < 0.001.

Since the response to mRNA vaccine correlates with the tumor immune microenvironment, the single-sample gene set enrichment analysis (ssGSEA) algorithm was employed to score 28 immune-related signatures to evaluate the enrichment of immune cell components in two immune subtypes across the TCGA and GSE54467 cohorts. Interestingly, most immune cells were more enriched in IS2 than in the IS1 subtype (**Figures 4A–D**). In addition, the evaluation of the immune status of the tumor microenvironment by the ESTIMATE algorithm indicated that the IS2 subtype had remarkably higher immune, stromal, and estimate scores than IS1 both in the TCGA and GSE54467 cohorts (**Figures 4E–J**). Hence, IS2 was identified as an immune-hot phenotype, while IS1 was an immune-cold phenotype. These suggest that SKCM patients in the IS1 subtype with immune-cold phenotype might be suitable candidates for the mRNA vaccines we studied.

### The immune landscape of SKCM

**Figure 5:**
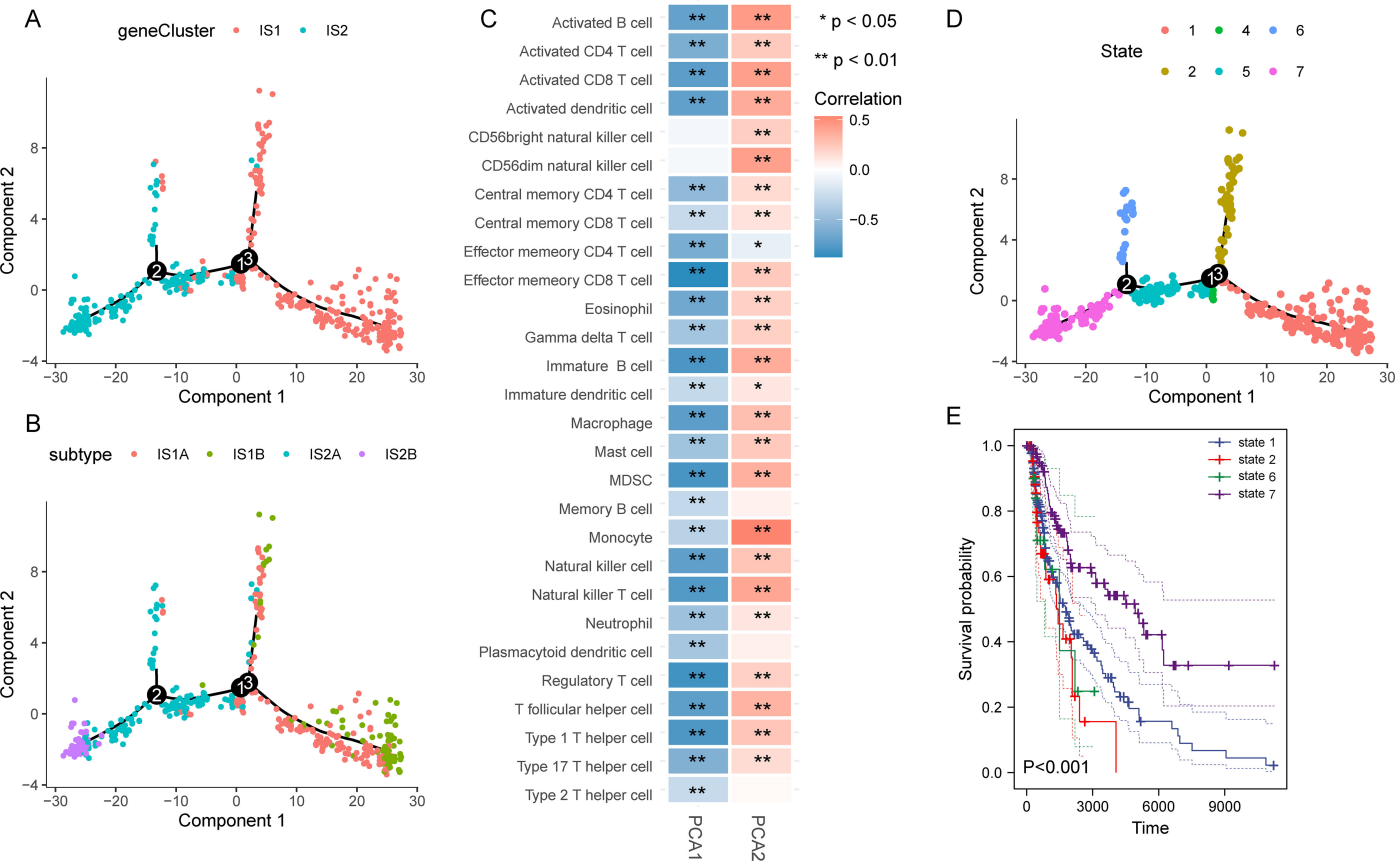
Construction of the immune landscape for SKCM. **(A)** Immune landscape of SKCM. Each point represents a patient, and the immune subtypes are color-coded. **(B)** Immune landscape of the subclusters of immune subtypes. **(C)** Heatmap of two principal components with 28 immune cell signatures. **(D, E)** Immune landscape of patients from three extreme locations **(D)** and their prognosis **(E)**.

We further constructed the immune landscape of individual SKCM patients by integrating the immune gene expression profiles (**Figure 5A**). Moreover, each immune subtype could be divided into two subgroups according to the distribution location in the immune landscape (**Figure 5B**). Principal component 1 was negatively correlated with most immune cells, while principal component 2 had a positive correlation with those immune cells besides effector memory CD4 T cell (**Figure 5C**). Then, the patients were divided into seven states and their survival curves showed the immune landscape had an excellent prognostic effect (**Figures 5D, E**). Collectively, the immune landscape we constructed could precisely identify the immune components of each patient and evaluate their prognoses, which contribute to developing personalized therapeutic cancer mRNA vaccines.

### Weighted gene co-expression network analysis of characteristic genes for SKCM immunotyping

**Figure 6:**
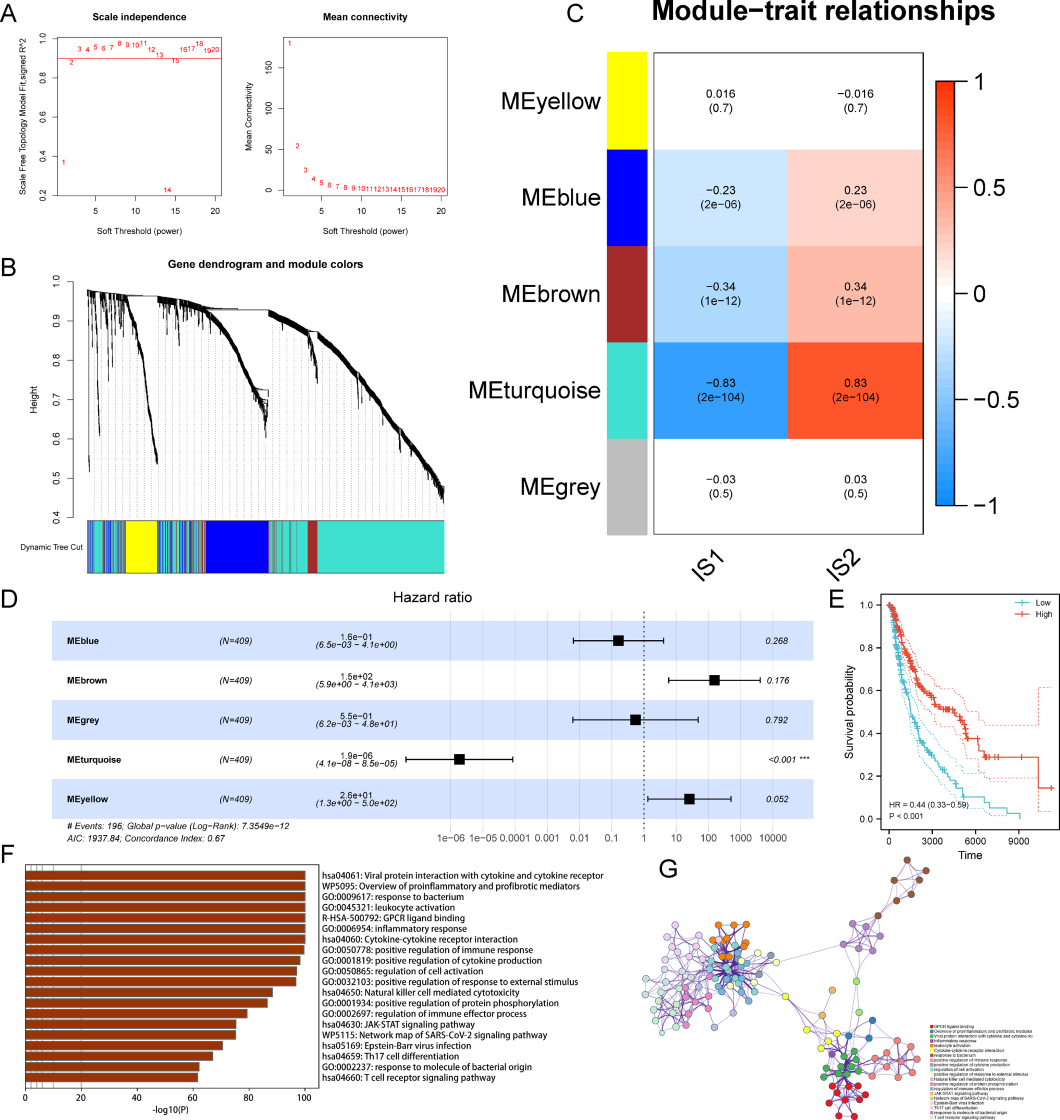
Identification of immune gene co-expression modules of SKCM. **(A)** Scale-free fit index (left) and mean connectivity (right) for various soft-thresholding powers. **(B)** Dendrogram of immune genes clustered based on the average-linkage hierarchy clustering method. **(C)** Correlations between modules and immune subtypes. **(D)** Prognostic analysis of 5 modules. **(E)** Survival analysis of patients with high and low scores for genes in the turquoise module. **(F)** Top 20 of GO and KEGG functional enriched pathways of genes in the turquoise module. **(G)** Metascape analysis of top 20 functional enriched pathways.

The WGCNA was employed to identify the genes associated with suitable vaccination clusters. In the scale-free network, the optimal soft threshold was chosen at 2 and determined by connectivity (**Figure 6A**). After calculating the eigengenes for each module, we picked five gene modules (**Figure 6B**). Next, the relationship between the five modules and immune subtypes was explored, and the close association between the turquoise module and IS2 was observed (**Figure 6C**). Besides, univariate Cox analysis revealed that the turquoise module had a distinct correlation with the prognosis of SKCM (**Figure 6D**). Patients with high gene expression scores in the turquoise module survived longer than those with low scores (**Figure 6E**). Genes in the turquoise module were listed in **Supplementary Table S2**. Functional enrichment analysis for genes from the turquoise module demonstrated immune response-related pathways were involved, such as inflammatory response, leukocyte activation, cytokine-cytokine receptor interaction, and positive regulation of immune response (**Figure 6F**). Additionally, metascape analysis revealed the relevance among these pathways and showed in **Figure 6G**. In summary, genes in the turquoise module might serve as the marker for selecting patients suitable for mRNA vaccine.

## Discussion

SKCM is one of the most aggressive skin malignancies originating from melanocytes with a poor prognosis. Although several therapies are available in treatment of SKCM, the prognosis is still unsatisfactory. Immunotherapy, including checkpoint inhibitors, targeted therapies, and antibody-drug conjugates, is the most modern treatment option for advanced SKCM and is continuing to develop (30). In particular, the application of mRNA vaccines is regarded as a promising immunotherapeutic strategy based on preclinical and clinical trials with impressive efficacy (21). Unfortunately, there are few studies on mRNA vaccines for SKCM. It is approved that bioinformatics analysis can accelerate the identification and selection of potential tumor antigens with high specificity and immunogenicity for mRNA vaccines by analyzing extensive datasets related to genetic expression and variation information. Therefore, in this study, we utilized bioinformatics analysis to discover neoantigens in SKCM and potential subpopulations for vaccination contributing to the development of personalized vaccines for each patient.

Recent reports demonstrated that the tumor-associated antigens are significantly overexpressed in cancers, which influences the specific immune cell response to personalized antigen immunization (31). Besides, alterations in genomes are closely correlated with the metastasis and development of SKCM (32). Therefore, aberrant expression and genetic alteration profiles were constructed to reveal targetable antigens that may be considered to treat SKCM. We then identified overexpressed five SKCM antigens (P2RY6, PLA2G2D, RBM47, SEL1L3, and SPIB) were associated with better prognosis and high infiltration of antigen-presenting cells. In addition, our RT-qPCR analysis showed high expression levels of P2RY6, PLA2G2D, SEL1L3, and SPIB in SKCM tissue, indicating these antigens were promising candidates for mRNA vaccine. However, the restricted sample size inherently limits the reliability. Consequently, we intend to augment our validation efforts in subsequent studies by integrating a larger cohort of samples and exploring the application of *in vitro* models to further substantiate our findings.

Although further clinical evaluation is required, present studies have shown the potential role of these genes in immunotherapy. P2RY6 is involved in modulating the immune response of multiple inflammatory diseases. It is also considered a critical positive regulator of skin tumorigenesis by the Hippo/YAP and Wnt/β-catenin signaling pathways (33). P2RY6 has been reported as a prognosis biomarker in lung adenocarcinoma patients and is associated with the immune microenvironment. These suggested that P2RY6 might be a novel target for immunotherapy in SKCM. Additionally, PLA2G2D is an immune- and metabolism-associated molecule identified as a biomarker to predict the prognosis of cervical squamous cell carcinoma. Patients with high expression levels of PLA2G2D exhibited notable high infiltration of immune cells, especially T cells and macrophages. PLA2G2D expression was positive with the expression of immune checkpoints and impacted the immune checkpoint blockade therapeutic efficacy (34). PLA2G2D and six other BRAF-associated genes were used to develop a prognostic signature for SKCM (35). Besides, RBM47 is a post-transcriptional regulator of RNA during tumor progression. It is a key mediator involved in the function of miR-25 in SKCM, suggesting that RBM47 is a promising target for treating SKCM (36). In addition, SEL1L3, with the other three genes, was utilized to build a signature that can predict survival and anti-CTLA4 immunotherapeutic responses (37). Moreover, SPIB is a transcription factor mainly expressed in mature B cells, plasma cell-like cells, and T cell precursor cells. It acts as a tumor suppressor and exerts its anti-colorectal cancer effect by activating the NF-kB and JNK signaling pathways through MAP4K1 (38).Taken together, these findings suggest that the five genes we screened have demonstrated potential as tumor antigens in various cancers, making them promising candidates for the development of future mRNA vaccines. Although these antigens show promise for vaccine development due to their aberrant tumor expression, their high expression in normal tissues may cause autoimmunity or immune-related side effects. For example, PLA2G2D is highly expressed in immune cells, and RBM47 in kidney and intestine (30, 32). Thus, future studies should focus on assessing their normal tissue expression to ensure tumor specificity and guide mRNA vaccine target selection.

Tumor heterogeneity is one of the major factors of the low effect of tumor vaccine. Only part of cancer patients gains benefits from mRNA vaccines according to the clinical trials (39). To investigate which subpopulation was suitable for vaccination, we identified two immune subtypes in the light of the immune gene profile. SKCM patients in IS1 exhibited better prognosis and a larger proportion in low grade than those in IS2. Furthermore, samples in IS2 displayed higher levels of TMB, mutation number and MSI. Different genetic mutation states were shown between subtypes IS1 and IS2. Specially, TNN, MUC16, BRAF, DNAH5 carried the highest frequency mutations. Reports demonstrated high-frequently mutations in TNN and MUC16 had better prognosis and immunotherapy efficacy than patients with the low-frequently mutations or the wild-type genes in cancer patients (40, 41). Previous research also found MUC16 had a higher mutation frequency in SKCM patients, which affect immune-related pathways and tumor-infiltrating immune cells resulting in better prognosis (42). Notably, about 50% of people whose SKCMs have a BRAF V600 mutation. BRAF V600 mutation-targeted therapies with Dabrafenib, have be accepted as a standalone treatment for advanced melanoma with this mutation (43). Therefore, these genetic mutations contribute to SKCM heterogeneity and follow take affect vaccine protection.

Importantly, the immune subtypes differed in the expressions of immune checkpoints and immune cell death modulators which could influence the efficacy of the mRNA vaccine. IS2 were associated with a high expression level of most modulators we examined. Recently studies suggested that the mRNA vaccine could be less effective in patients with increased expression of immune checkpoints, and more effective in those with upregulation of immune cell death modulators (16). Therefore, patients in these two immune subtypes generate different immune responses after mRNA vaccination.

We further investigated the cellular and molecular characteristics of the immune subtypes. IS1 was discovered to have an immunologically cold phenotype with low expression of immune signatures, while IS2 was characterized by an immune “hot” phenotype. Patients in the immunologically cold phenotype are considered to be lack of antigen-presenting cells and tumor antigens, leading to T cell anergy and insensitivity to antigen activation (44, 45). Interestingly, the immune “hot” tumor displayed higher tumor sensitivity to immunotherapy because of high levels of infiltrating immune cells (46). Hence, the application of mRNA vaccines in immune “cold” patients may be an appropriate therapeutic option to convert tumors into immunologically cold phenotype.

The further immune landscape of SKCM revealed the heterogeneity among individuals as well as within the same immune subtypes. Next, the patients were divided into seven states, and observed patients in states 1, 2, and 6 had significantly worse survival than those in state 7 due to the relative immune-suppressive tumor microenvironment. WGCNA discovered the most relevant characteristic gene module. The functional annotation manifested that genes in this key module were enriched in immune-related pathways, supporting the follow-up exploration of the potential biological mechanism of subtypes.

Despite the identification of potential SKCM antigens, the development of personalized treatment using SKCM mRNA vaccines remains a formidable challenge in clinical practice. The application of tumor mRNA is confronted with numerous obstacles that include difficulties in evaluating anti-tumor immunity induced *in vivo*, immune evasion and resistance, as well as the personalization of medication for individual patients (47, 48). To address these challenges, a more comprehensive approach to subtyping can be established by integrating multi-omics analyses, including genomics, transcriptomics, morphology, proteomics, metabolomics, and immune subtypes, which can guide the precise diagnosis and treatment of tumors (49). Furthermore, an in-depth analysis of the molecular mechanisms underlying the *in vivo* application of mRNA vaccines will be conducted, with the aim of exploring the mechanisms of anti-tumor immunity, immune evasion, and resistance.

This study provides valuable insights into the relationship between potential tumor antigens and immune responses, but it has notable limitations. The analysis primarily relies on bioinformatics methods, which may not encompass all potential antigen outcomes. While specific antigens have been identified, further validation through *in vitro* and animal studies is essential to confirm their immunogenicity and clinical relevance. Future work will focus on detailed experiments to assess the immune responses elicited by these antigens in various contexts, evaluating their potential as effective mRNA vaccine candidates. This approach will enhance our understanding of antigen immunogenicity and contribute to optimizing vaccine design.

## Conclusions

In conclusion, through comprehensive bioinformatics analysis, P2RY6, PLA2G2D, RBM47, SEL1L3, and SPIB were determined to be potential antigens for SKCM mRNA vaccines, specifically for patients with IS1 tumors. These findings provide a novel insight for the anti-SKCM mRNA vaccine and defining suitable patients for vaccination.

## Data Availability

The original contributions presented in the study are included in the article/**Supplementary Material**. Further inquiries can be directed to the corresponding authors.

## Supplementary material

Supplementary Table 1 Primer sequences for RT-qPCR.

Supplementary Table 2 Gene list of the turquoise module.
