# Supplementary material for: Bioinformatics analysis reveals novel tumor antigens and immune subtypes of skin cutaneous melanoma contributing to mRNA vaccine development
Source: Front Immunol. 2025 Feb 24;16:1520505. doi: 10.3389/fimmu.2025.1520505 (PMC11891200; doi:10.3389/fimmu.2025.1520505)
Supplement: Supplementary file 3 [file DataSheet1.docx]

**Supplementary Figure 1**

**
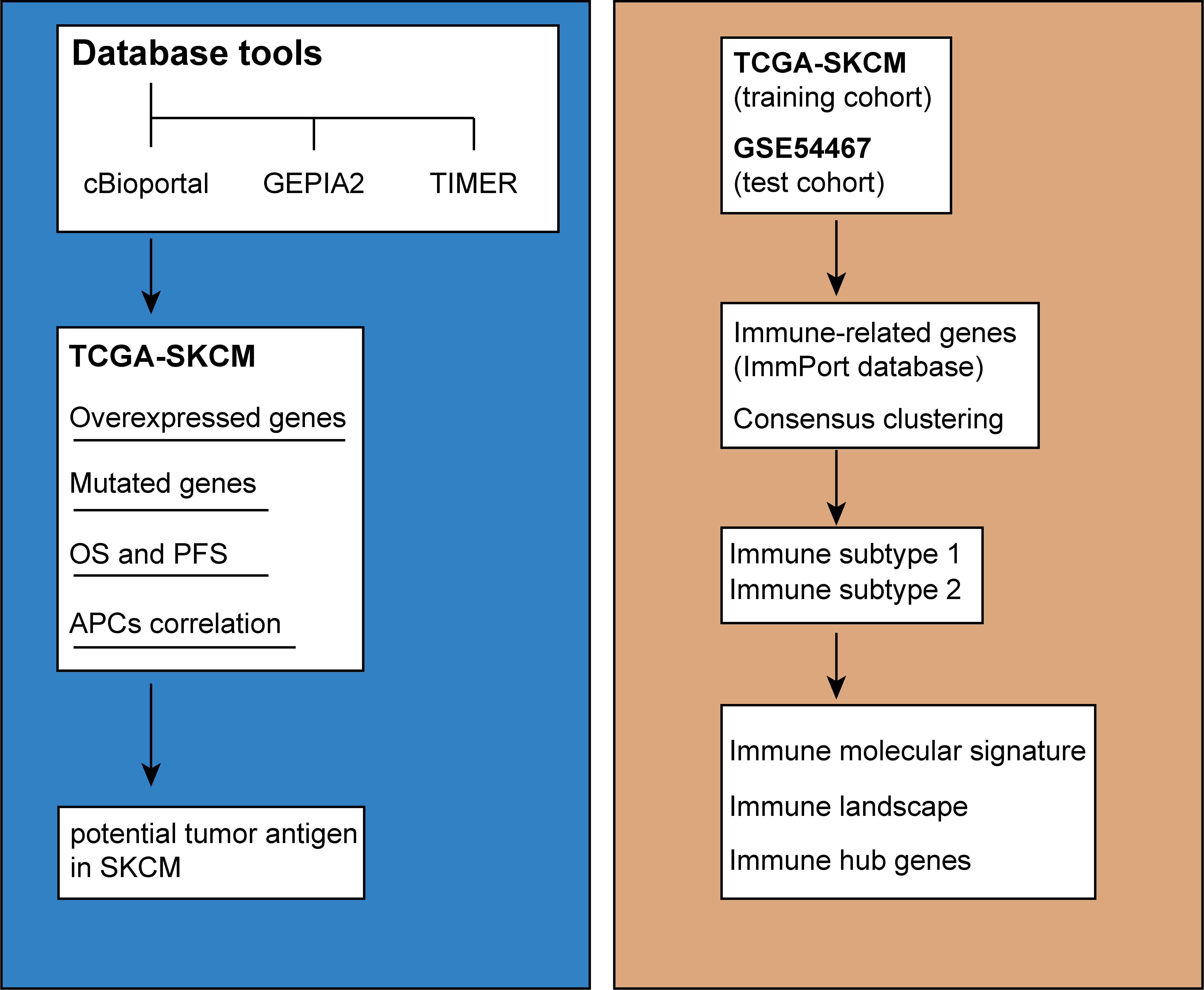
**

**Supplementary Figure 1. The workflow of this study.**

**Supplementary Figure 2**

**
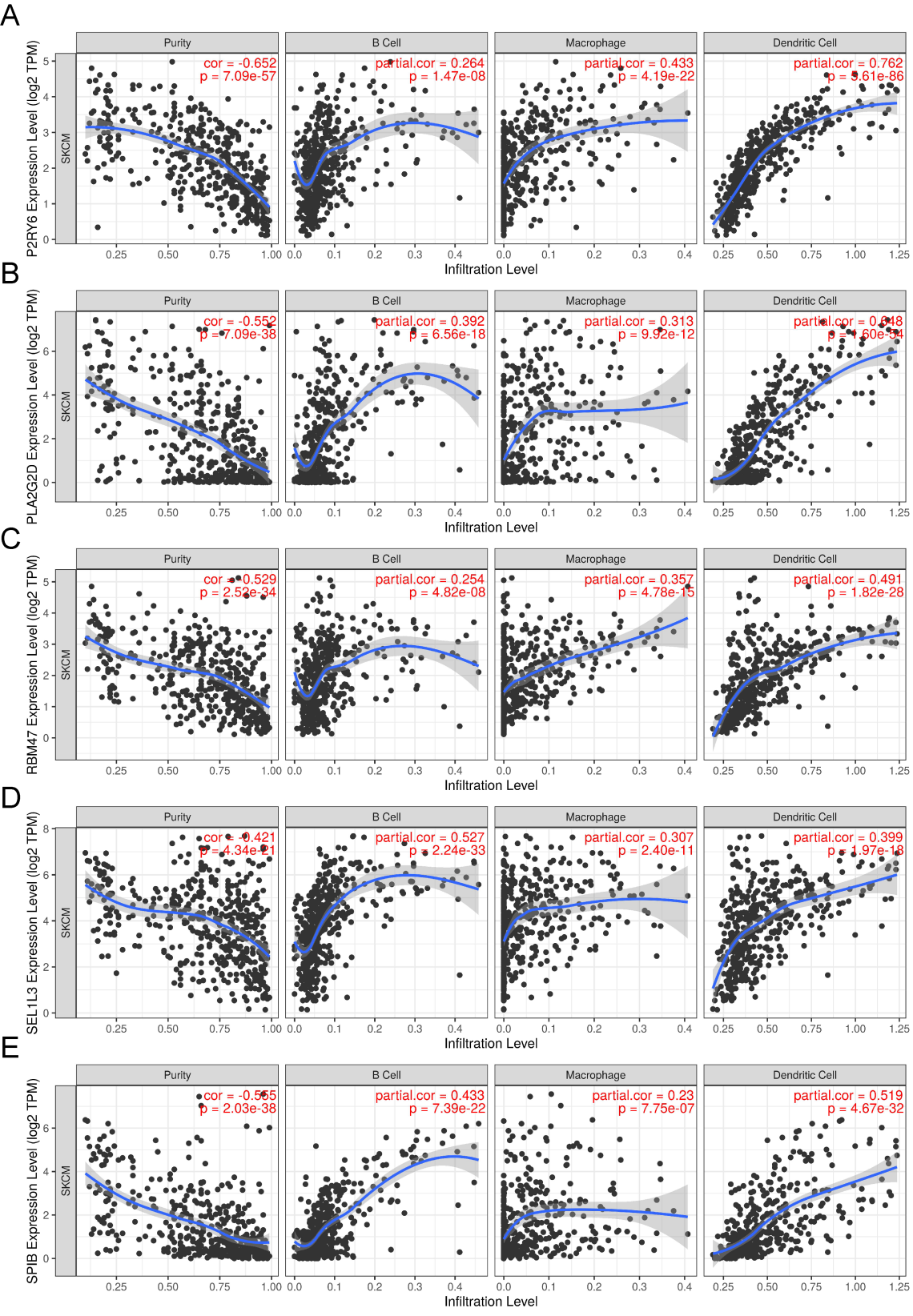
**

**Supplementary Figure 2. The relevance between five potential antigens and antigen-presenting cells.**

Correlation of P2RY6 **(A)**, PLA2G2D **(B)**, RBM47 **(C)**, SEL1L3 **(D)**, SPIB **(E)** expression with the infiltration of B cells, macrophages, and dendritic cells.

**Supplementary Figure 3**

**
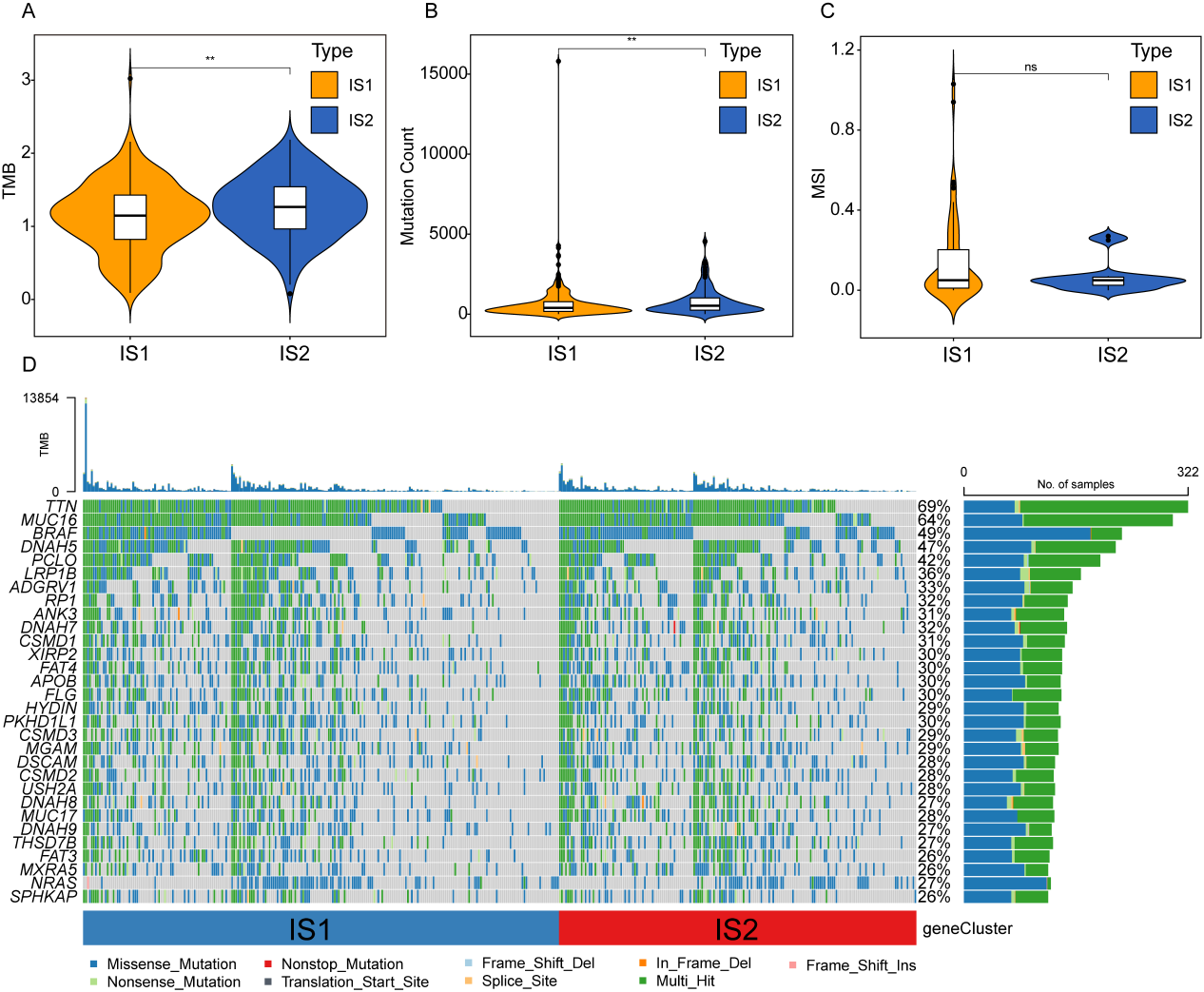
**

**Supplementary Figure 3. Association of immune subtypes with mutation status.**

**(A-C)** TMB **(A)**, mutation count **(B)**, and MSI **(C)** were evaluated in two immune subtypes in TCGA SKCM cohort. **(D)** Thirty highly mutated genes in the SKCM immune subtypes. * p < 0.05, ** p < 0.01.

**Supplementary Figure 4**

**
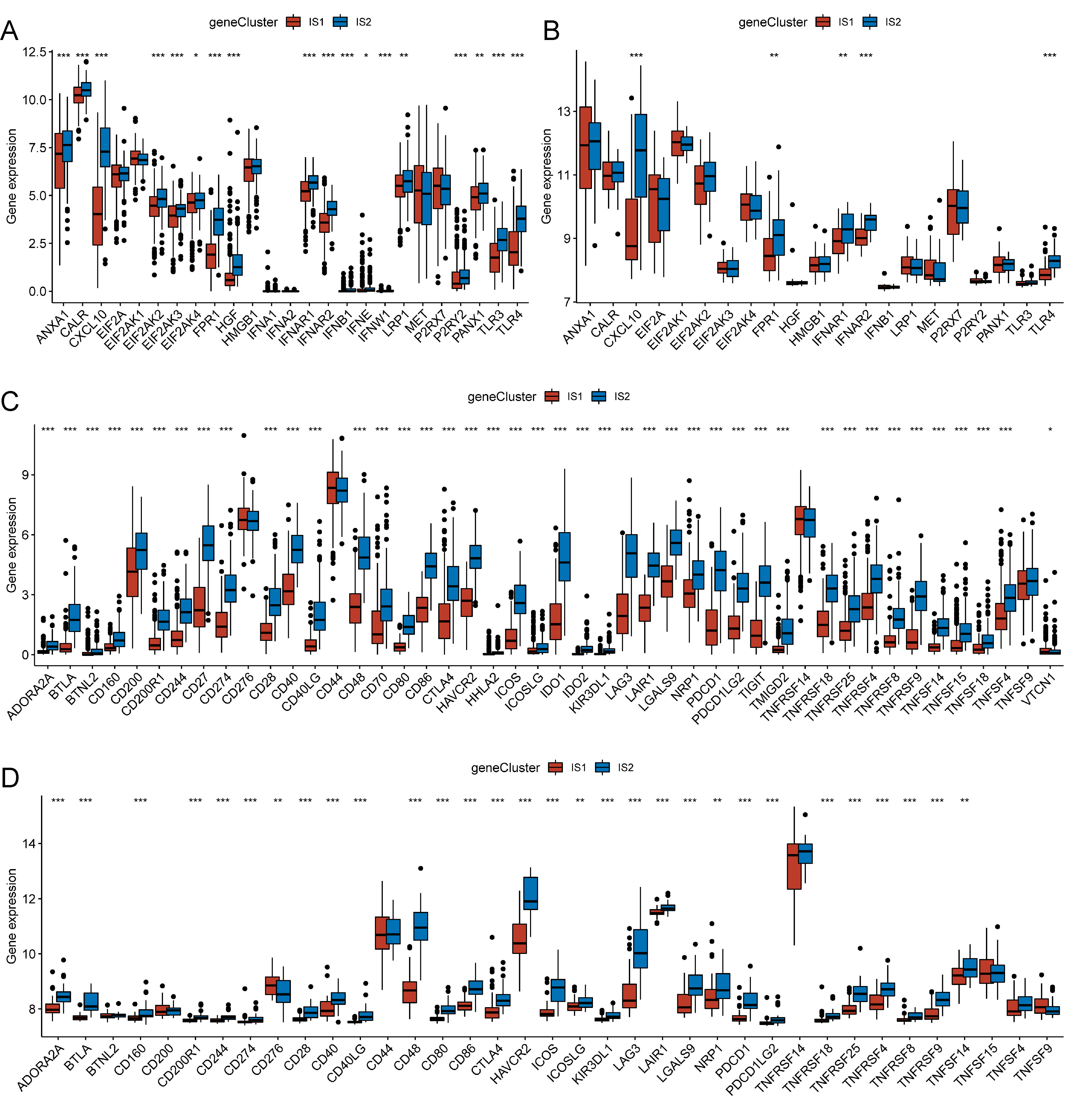
**

**Supplementary Figure 4. Relationship of immune subtypes with ICPs and ICD modulators**

**(A-B)** Boxplots exhibiting the differential expression of ICDs between IS1 and IS2 subtypes in the TCGA **(A)** and GSE54467 **(B)** cohorts. **(C-D)** Differential expression of ICPs modulator genes between the immune subtypes in TCGA **(C)** and GSE54467 **(D)** cohorts.
